# Usefulness of Molecular Diagnostic Kits for Detecting *Helicobacter pylori* Infection in Patients Receiving Proton Pump Inhibitors and Potassium‐Competitive Acid Blockers

**DOI:** 10.1155/bmri/2610573

**Published:** 2026-03-14

**Authors:** Kentaro Wakamatsu, Tsutomu Nishiyama, Kunio Yamada, Koya Yamada, Shohei Mitsumata, Tetsuharu Ikeda, Kouta Katsuki, Tomoki Takeyama, Ruriko Kiyotani, Izumi Fukui, Masayuki Kawasaki, Zenzo Nagasawa, Nobuhiko Nagata, Satomi Asai, Takashi Hisabe

**Affiliations:** ^1^ Department of Respiratory Medicine, National Hospital Organization Omuta National Hospital, Fukuoka, Japan, hosp.go.jp; ^2^ Department of Gastroenterology, Nishiyama Clinic, Fukuoka, Japan; ^3^ Department of Internal Medicine, Arao Clinic, Kumamoto, Japan; ^4^ Department of General Thoracic Surgery, Breast and Pediatric Surgery, Fukuoka University School of Medicine, Fukuoka, Japan, fukuoka-u.ac.jp; ^5^ Department of Medical Laboratory Science, Graduate School of Health and Welfare Sciences, International University of Health and Welfare Graduate School, Fukuoka, Japan; ^6^ Department of Respiratory Medicine, Fukuoka Sanno Hospital, Fukuoka, Japan; ^7^ Department of Laboratory Medicine, Tokai University School of Medicine, Isehara, Kanagawa, Japan, u-tokai.ac.jp; ^8^ Department of Gastroenterology, Fukuoka University Chikushi Hospital, Fukuoka, Japan

**Keywords:** clarithromycin resistance, gene mutations, *Helicobacter pylori* infection, molecular diagnostic kit, potassium-competitive acid blocker, proton pump inhibitor, Smart Gene

## Abstract

**Background:**

*Helicobacter pylori* infection is associated with gastric cancer; therefore, therapies that eradicate *H. pylori* are crucial for its prevention. This study investigated the effects of clarithromycin (CAM)‐resistance gene mutations on the success rate of *H. pylori* eradication and the usefulness of the Smart Gene *H. pylori* G (Mizuho Medy Co. Ltd., Saga, Japan) molecular diagnostic kit for *H. pylori* infection, which detects CAM‐resistance mutations.

**Methods:**

This study enrolled patients who underwent esophagogastroduodenoscopy, during which intragastric fluid was collected. The Smart Gene kit was used to diagnose *H. pylori* infection and CAM‐resistance mutations, and results were compared with those of the conventional urea breath test (UBT) and real‐time polymerase chain reaction (PCR) results.

**Results:**

The study included 253 patients. *H. pylori* infection was confirmed by real‐time PCR in 23 (9.1%) patients, and 99 (39.1%) patients were receiving a proton pump inhibitor (PPI) or potassium‐competitive acid blocker (P‐CAB). The sensitivities and specificities of the Smart Gene test and UBT were 87.0% and 82.6%, as well as 99.6% and 93.0%, respectively. PPI/P‐CAB use did not affect the distribution of *H. pylori* DNA levels measured by real‐time PCR, but the UBT values were significantly lower in the medicated group than in the unmedicated group (*p* < 0.05). Thirteen patients underwent treatment to eradicate *H. pylori*, with successful eradication in 11 (84.6%). The Smart Gene test identified three patients with CAM‐resistance mutations. One patient was successfully treated with a metronidazole‐based regimen, whereas CAM‐based eradication therapy failed in two patients.

**Conclusions:**

The Smart Gene molecular diagnostic test using intragastric fluid was useful for patients requiring PPI/P‐CAB to manage gastrointestinal symptoms. It could also confirm an *H. pylori* infection and CAM‐resistance mutations within 1 h of testing. This diagnostic test may help reduce side effects and the emergence of resistant strains due to unnecessary eradication therapy.

## 1. Introduction

*Helicobacter pylori* is a helical, gram‐negative rod with multiple flagella that was first isolated and cultured from the stomachs of patients with gastritis and gastric ulcers in 1982 [1]. *H. pylori* infection causes various upper gastrointestinal diseases, such as atrophic gastritis, gastric and duodenal ulcers, gastric cancer, gastric mucosa‐associated lymphoid tissue lymphoma, and gastric hyperplastic polyps [2], and is associated with gastric cancer [3]. The International Agency for Research on Cancer of the World Health Organization has stated that *H. pylori* causes gastric cancer and recommended appropriate policies to prevent gastric cancer, including eradication therapy [4]. In Japan, *H. pylori* eradication therapy for chronic gastritis has been covered by insurance since 2013. Since then, *H. pylori* eradication therapy has been widely implemented for the primary prevention of gastric cancer, and the number of gastric cancer‐related deaths has been decreasing [5]. *H. pylori* eradication in Japan has a high‐economic health benefit, with reduced health costs and benefits for patients of all ages [6].

Clarithromycin (CAM)‐resistant *H. pylori* reduces the success rate of eradication therapy [2]. The Japanese guidelines recommend performing antimicrobial susceptibility testing and that the combination with the highest eradication rate should be used for treatment [2]. However, antimicrobial susceptibility testing based on bacterial cultures requires a minimum of 1 week of testing period. In addition, bacterial cultures are not always possible. Therefore, a test method that can rapidly detect *H. pylori* infection and CAM resistance is desired.

In 2022, molecular diagnostic tests to detect *H. pylori* and CAM‐resistant gene mutations were covered under insurance in Japan. This molecular diagnostic kit, Smart Gene *H. pylori* G (Mizuho Medy Co. Ltd., Saga, Japan), can diagnose *H. pylori* infection and CAM‐resistance gene mutations within an hour using intragastric fluid collected during esophagogastroduodenoscopy (EGD) without additional invasive procedures. Moreover, intragastric fluid can be collected safely and without the risk of hemorrhage, unlike gastric mucosal biopsies. Previous studies have reported a high concordance rate among the Smart Gene test, conventional diagnostic methods, and antimicrobial susceptibility testing for *H. pylori* infection. This makes it an excellent alternative diagnostic method that saves physicians time by avoiding eradication failures due to CAM resistance [7–9].

The effects of proton pump inhibitor (PPI) or potassium‐competitive acid blocker (P‐CAB) medication on *H. pylori* diagnostic test results are a major concern. PPIs affect conventional diagnostic methods for *H. pylori* infection, especially the urea breath test (UBT) [10]. Patients with suspected *H. pylori* infection often present with gastrointestinal symptoms, and withdrawing from PPI/P‐CAB medications for diagnostic infection testing can be problematic for managing patient symptoms. A recent study reported that the stool antigen test, which is not based on urease activity, remained antigenic, even when PPIs were taken [11]. Molecular diagnostic tests using gastric samples, such as gastric juice and gastric biopsy, are also excellent test methods minimally influenced by PPI [12, 13]. Hence, the 2024 Japanese guidelines state that tests to detect the urease activity of *H. pylori* require PPI/P‐CAB withdrawal, whereas molecular diagnostic and stool antigen tests that directly detect *H. pylori* can be performed without PPI/P‐CAB withdrawal [2].

The Smart Gene molecular diagnostic test kit is now available for clinical use. Therefore, this study evaluated its usefulness for detecting *H. pylori* in patients undergoing PPI/P‐CAB and determining CAM‐resistant mutations in patients who underwent *H. pylori* eradication therapy based on eradication outcomes.

## 2. Materials and Methods

### 2.1. Study Design and Patient Background

This study included patients who underwent EGD at the NHO Omuta Hospital, Nishiyama Clinic, and Arao Clinic between March 2023 and April 2024. All patients provided written consent and were enrolled regardless of whether they were taking PPI/P‐CAB. Patients were initially tested using UBT as a conventional diagnostic test for *H. pylori* infection. Subsequently, intragastric fluid was collected from the EGD. Two *H. pylori* molecular diagnostic tests, the Smart Gene test and real‐time polymerase chain reaction (PCR), were performed using intragastric fluid. To minimize the burden on patients, a gastric biopsy was not collected for this study; therefore, the rapid urease test (RUT) and culture test were not performed. The Ethics Committee of the National Hospital Organization Omuta Hospital approved this study (Approval No. 4‐36), which was conducted following the principles of the Declaration of Helsinki.

### 2.2. Effect of PPI/P‐CAB Medication on *H. pylori* Detection

The effect of PPI/P‐CAB medication on the diagnosis of *H. pylori* infection was evaluated using patients positive for *H. pylori* infection based on real‐time PCR. The patients were stratified based on whether they were taking PPI/P‐CAB. The distribution of UBT measurements and *H. pylori* DNA levels was compared and evaluated.

### 2.3. Sample Collection Timing of Intragastric fluid

Intragastric fluid was collected during EGD in a volume of at least 5 mL, as previously reported [7, 9]. During gastric fluid collection, a sterilized collection kit was connected to the adequately disinfected suction port of the endoscope, and appropriate measures were taken to prevent contamination. Collection was performed either at the start of EGD (Collection Time 1), with fluid obtained immediately after the start of the procedure, or later during EGD (Collection Time 2), when the gastric mucosa at the suspected site of *H. pylori* infection was sprayed with water and the resulting washings (20–50 mL) containing mucus were collected. In both cases, the intragastric fluid was collected prior to staining and the collection of the gastric mucosal biopsy. The timing of intragastric fluid collection was stratified into Collection Times 1 and 2 and evaluated based on the distribution of *H. pylori* DNA levels.

### 2.4. *H. pylori* Eradication Results

Patients diagnosed with *H. pylori* infection and treated for eradication were evaluated. The eradication outcome was assessed based on the Smart Gene CAM‐resistance gene mutation test and eradication regimen outcomes.

### 2.5. Smart Gene *H. pylori* G and Real‐Time PCR Testing

The commercially available molecular diagnostic kit, Smart Gene *H. pylori* G (Mizuho Medy Co. Ltd., Saga, Japan), was used to detect mutations in the 23S rRNA domain V region associated with *H. pylori* infection and CAM resistance. The test was performed using the dedicated Smart Gene measuring instrument with intragastric fluid as the test sample, per the manufacturer′s instructions. As a control, real‐time PCR of the *H. pylori* 16S rRNA gene, a different target from the Smart Gene, was performed based on a previous report [14]. In brief, we extracted DNA from 200 *μ*L of intragastric fluid with the QIAamp DNA Mini Kit (QIAGEN GmbH, Hilden, Germany) to obtain 150 *μ*L of purified DNA. Real‐time PCR was performed using the Thermal Cycler Dice Real Time System III instrument (Takara Bio Inc., Shiga, Japan) and TB Green Premix Dimer Eraser Perfect Real Time reagent (Takara Bio Inc.) under the following PCR conditions: preheating at 95°C for 30 s, and 50 cycles at 95°C for 5 s, 55°C for 30 s, and 72°C for 30 s. The PCR amplicons were confirmed using melting curve analysis.

### 2.6. Statistical Analysis

Nonparametric analyses were performed because the data were not normally distributed. Values were presented as medians and interquartile ranges. The 95% confidence intervals for sensitivity and specificity were estimated using the Clopper–Pearson method. The chi‐square test was used to compare *H. pylori*‐infected and noninfected groups. Fisher′s exact test was applied to evaluate the effects of PPI/P‐CAB medication use and sample collection timing, because of the small number of patients in some strata. Statistical significance was set at *p* < 0.05. All statistical analyses were performed using Excel (Bell Curve for Excel Version 3.21; Social Information Service Co. Ltd., Tokyo, Japan).

## 3. Results

### 3.1. Patient Background

Table 1 presents the patients′ backgrounds. In total, 253 patients were enrolled, of which 23 patients (9.1%) had confirmed *H. pylori* infection using real‐time PCR, and 99 patients (39.1%) were on PPI/P‐CAB medication. Sex, medication, and timing of intragastric fluid collection did not differ among the groups.

**Table 1:** Patient backgrounds.

	Real‐time PCR		
	Positive	Negative	*p*
Number of patients	23	230	—
Age			
Median (IQR)	74 (62–83)	71 (58–78)	0.0943
Gender			
Female (%)	10 (45%)	106 (46%)	0.8108
PPI/P‐CAB use			
Current medication (%)	9 (39%)	90 (39%)	1.000
Antimicrobial use			
Current medication (%)	1 (4%)	4 (2%)	0.3914
Collection of intragastric fluid			
Initial collection (%)	16 (70%)	135 (59%)	0.3109

Abbreviations: IQR, interquartile range; PCR, polymerase chain reaction; PPI, proton pump inhibitor; P‐CAB, potassium‐competitive acid blocker.

### 3.2. Evaluation of Diagnostic Methods for *H. pylori* Infection

Table 2 presents the results of the diagnostic methods used to detect *H. pylori* infection. Compared with real‐time PCR, the sensitivity and specificity were 82.6% and 93.0%, as well as 87.0% and 99.6% for the UBT and Smart Gene tests, respectively. Table 3 presents the cases of discordance in the diagnostic methods for *H. pylori* infection regarding real‐time PCR. UBT produced four false‐negative results and 16 false‐positive results, and the Smart Gene test produced three false‐negative results and one false‐positive result.

**Table 2:** Comparison of the diagnostic methods for *H. pylori* infections.

Real‐time PCR	UBT		Smart Gene	
	Positive	Negative	Positive	Negative
Positive	19	4	20	3
Negative	16	214	1	229
Sensitivity	82.6% (61.2%–95.0%)		87.0% (66.4%–97.2%)	
Specificity	93.0% (88.9%–96.0%)		99.6% (97.6%–100%)	

*Note:* Data in parentheses are 95% confidence intervals.

Abbreviation: UBT, urea breath test.

**Table 3:** List of the mismatched cases.

Real‐time PCR (copies/*μ*L)	Patient background		Diagnostic method	
	PPI/ P‐CAB	Antimicrobial	UBT (%)	Smart Gene
Pos (2.5×10) Pos (3.2×10^2^) Pos (4.9×10^4^) Pos (1.4×10) Pos (3.1×10) Pos (1.2×10^2^)	Medication Medication Medication None Medication None	None None None None None None	Neg Neg Neg Neg Pos (4.0) Pos (16.4)	Neg Pos Pos Pos Neg Neg
Neg Neg Neg Neg Neg Neg Neg Neg Neg Neg Neg Neg Neg Neg Neg Neg	None Medication Medication Medication Medication Medication None None Medication None None None None None Medication Medication	None None None None None None None None None None None None None None None None	Pos (2.9) Pos (8.5) Pos (3.9) Pos (6.6) Pos (2.6) Pos (10.6) Pos (11.3) Pos (3.3) Pos (4.4) Pos (13.4) Pos (3.7) Pos (3.9) Pos (2.5) Pos (2.8) Pos (8.6) Pos (3.6)	Neg Neg Neg Neg Neg Neg Neg Neg Neg Neg Neg Neg Neg Neg Neg Pos

Abbreviations: Neg, negative; Pos, positive; PPI, proton pump inhibitor; P‐CAB, potassium‐competitive acid blocker; UBT, urea breath test.

### 3.3. Effect of PPI/P‐CAB Medication on the Diagnosis of *H. pylori* Infection

Figure 1 shows the effects of PPI/P‐CAB on the diagnosis of *H. pylori* infection. As quantitative values for the Smart Gene were unavailable, its effect on the molecular diagnostic methods was evaluated using real‐time PCR. Of the 23 patients with positive real‐time PCR results, nine were on PPI/P‐CAB medication. Of the 35 UBT‐positive patients, 14 were treated with PPI/P‐CAB. In the PPI/P‐CAB group, the measured UBT was significantly lower than that in the nonmedicated group (*p* < 0.05).

Figure 1Effect of PPI and P‐CAB medication on *Helicobacter pylori* infection diagnostic methods. Effect on (a) UBT and (b) real‐time PCR. The measurement distributions are presented using box plots. The centerlines represent the medians. The box limits indicate the 25th and 75th percentiles. The whiskers extend 1.5 times the interquartile range from the 25th and 75th percentiles. Circles represent outliers. PPI, proton pump inhibitor; P‐CAB, potassium‐competitive acid blocker; UBT, urea breath test; PCR, polymerase chain reaction.

**Figure:**
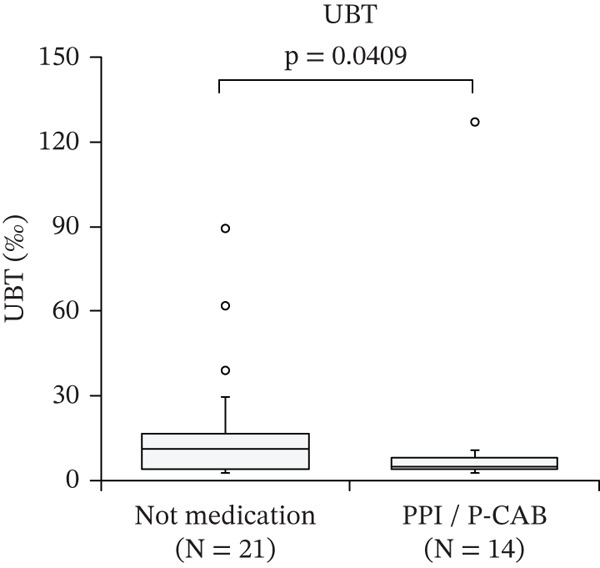
(a)

**Figure:**
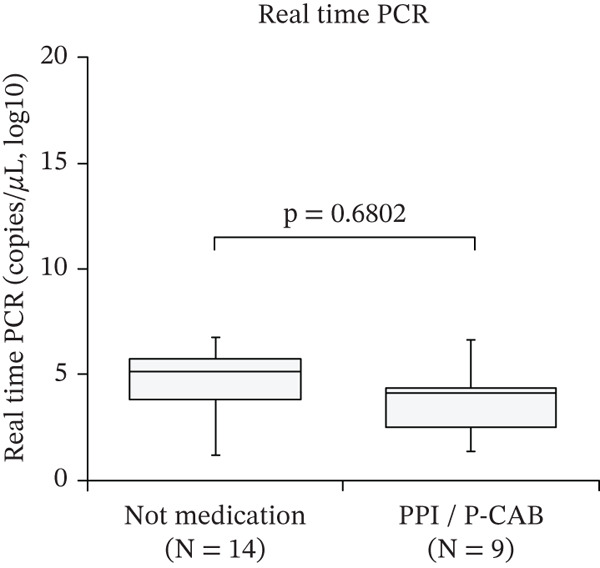
(b)

### 3.4. Effect of Intragastric Fluid Collection Timing on the *H. pylori* Molecular Diagnostic Method

**Figure 2:**
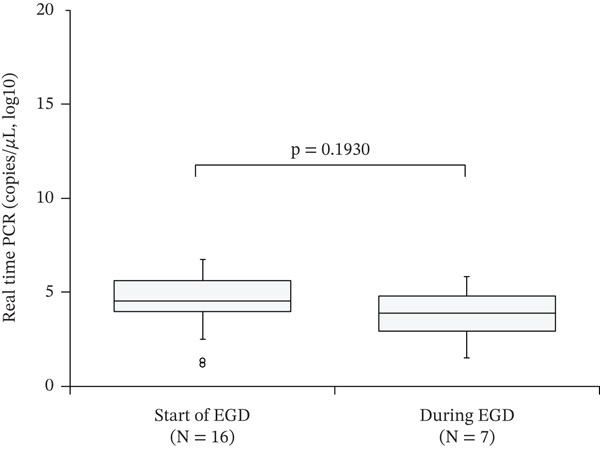
Effect of intragastric fluid collection timing on PCR‐based testing. The distribution of *Helicobacter pylori* DNA copies by real‐time PCR is presented using box plots. The centerlines represent medians. The box limits indicate the 25th and 75th percentiles. The whiskers extend 1.5 times the interquartile range from the 25th and 75th percentiles. Circles represent outliers. PCR, polymerase chain reaction; EGD, esophagogastroduodenoscopy.

Figure 2 shows the effect of intragastric fluid collection time on the *H. pylori* molecular diagnostic method. Of the 23 real‐time PCR‐positive cases, 16 were collected at the start of EGD. The *H. pylori* DNA quantified using real‐time PCR did not differ based on the sampling time (*p* = 0.1930).

### 3.5. *H. pylori* Eradication Therapy Outcomes

Table 4 presents the results of the *H. pylori* eradication therapy. Thirteen patients were treated for *H. pylori* infection, of which six cases had no CAM‐resistance mutations based on the Smart Gene test and were successfully eradicated. CAM‐resistance mutations were identified in three cases: one patient was successfully treated using a metronidazole (MNZ)‐based regimen, whereas treatment failed in two patients using the CAM‐based regimen.

**Table 4:** Results of the eradication treatment.

Diagnostic method		Eradication	
UBT	Smart Gene (CAM‐resistant mutation)	Regimen	Result
Pos	Neg	PPI/AMPC/MNZ	Success
Pos	Neg	P‐CAB/AMPC/CAM	Success
Pos	Neg	PPI/AMPC/CAM	Success
Pos	Neg	PPI/AMPC/CAM	Success
Pos	Pos (no mutation)	PPI/AMPC/CAM	Success
Pos	Pos (no mutation)	PPI/AMPC/CAM	Success
Pos	Pos (no mutation)	P‐CAB/AMPC/CAM	Success
Pos	Pos (no mutation)	P‐CAB/AMPC/CAM	Success
Pos	Pos (no mutation)	PPI/AMPC/CAM	Success
Pos	Pos (no mutation)	P‐CAB/AMPC/CAM	Success
Neg	Pos (mutation)	P‐CAB/AMPC/MNZ	Success
Pos	Pos (mutation)	PPI/AMPC/CAM	Failure
Pos	Pos (mutation)	PPI/AMPC/CAM	Failure

Abbreviations: AMPC, amoxicillin; CAM, clarithromycin; MNZ, metronidazole; Neg, negative; Pos, positive; PPI, proton pump inhibitor; P‐CAB, potassium‐competitive acid blocker; UBT, urea breath test.

## 4. Discussion

Testing for antimicrobial susceptibility in *H. pylori* infection and selecting an appropriate eradication regimen is crucial. Smart Gene *H. pylori* G is a new diagnostic kit for *H. pylori* infection that can detect CAM‐resistance mutations on the day of EGD. From a practical standpoint, stool antigen testing and the UBT remain well‐established and cost‐effective methods for routine *H. pylori* diagnosis; however, both are susceptible to the effects of acid‐suppressive therapy and often require treatment interruption. The Smart Gene system requires an initial equipment cost of approximately $5000 and a per‐test cost of around $30. Its assay procedure is relatively simple, with minimal hands‐on time [7], allowing integration into existing clinical laboratory workflows. Accordingly, rather than replacing established tests, Smart Gene may be best positioned as a complementary diagnostic option for patients in whom discontinuation of PPI or P‐CAB therapy is not feasible. This study clarified the effects of PPI/P‐CAB on this newly approved molecular diagnostic test using intragastric fluid.

In Japan, *H. pylori* infection rates vary widely by age group, with rates of approximately 40% among adults but less than 10% among those born in 1998 and later [15]. The *H. pylori* infection rate in Japan is presumably declining among older adults owing to widespread eradication therapy.

This study compared the diagnostic capacity of the Smart Gene test with UBT (a conventional method) and real‐time PCR (control method). We found that one patient had a negative real‐time PCR result but positive UBT and Smart Gene results, and three physicians strongly suspected *H. pylori* infection based on endoscopic findings, suggesting a possible false‐negative real‐time PCR result. UBT showed more false‐positive results than the Smart Gene test, and it was necessary to confirm UBT results near the cutoff value with endoscopic findings. Hence, the Smart Gene test was highly specific. In this study, the UBT yielded a relatively high number of false‐positive results compared with previous reports, where sensitivity and specificity generally exceeded 90%. Many of the false‐positive cases in our cohort showed values close to the cutoff level. As described above, endoscopic findings were often necessary to confirm the final diagnosis in these cases. A well‐recognized cause of UBT false positivity is the urease activity of oral bacteria. In addition, given the relatively high age of our study population, we speculate that inappropriate intake of the test meal might also have contributed to inaccurate UBT results. On the other hand, the sensitivity of real‐time PCR in our study did not appear to be excessively low when compared with infection diagnoses based on endoscopic evaluation.

According to the 2024 Japanese guidelines [2], stool antigen testing, which directly detects *H. pylori*, can be performed without discontinuation of PPI/P‐CAB therapy. However, PPI/P‐CAB administration is known to affect certain diagnostic methods, particularly UBT [10]. Therefore, we also evaluated the effect of PPI/P‐CAB on molecular diagnostics using intragastric fluid. We found that PPI/P‐CAB medication did not affect the distribution of *H. pylori* DNA in the intragastric fluid. In contrast, UBT values were significantly lower in medicated patients than in unmedicated patients. In this study, the type and duration of PPI/P‐CAB therapy were not evaluated, which represents an important limitation. Therefore, although our findings suggest that Smart Gene may be less affected by acid‐suppressive therapy than UBT, its clinical usefulness in patients requiring PPI/P‐CAB therapy should be interpreted with caution and warrants further investigation that accounts for medication type and duration.

The timing of intragastric fluid collection did not affect the Smart Gene results or the distribution of *H. pylori* DNA. Both collection times were suitable for testing. However, as a preventative measure, saliva and washing fluids, especially those from the esophagus, should be avoided because they may contain only low levels of *H. pylori* [9]. Collecting specimens from the deeper layers of the gastric body, rather than only from the upper layers of the gastric reservoir, is desirable.

The Japanese guidelines recommend drug susceptibility testing and selecting an appropriate eradication regimen to ensure *H. pylori* eradication. The standard primary eradication regimen is triple therapy with PPI or P‐CAB, amoxicillin, and CAM. The standard secondary eradication regimen is triple therapy in which CAM is replaced with MNZ [2]. Primary eradication failure is largely due to *H. pylori*′s resistance to CAM; thus, antimicrobial susceptibility testing is particularly important when selecting an eradication regimen. In this study, 13 of 28 patients diagnosed with *H. pylori* infection were treated, and 11 (84.6%) reported successful eradication. Three patients with CAM‐resistant mutations identified using the Smart Gene test were successfully treated with an MNZ‐based regimen, whereas eradication failed in two patients treated with CAM‐based regimens. These few cases illustrate how resistance information obtained using Smart Gene may inform treatment selection; however, they do not demonstrate improved eradication outcomes within this cohort. Recent evidence suggests that the therapeutic landscape of *H. pylori* eradication is rapidly evolving. A large systematic review and meta‐analysis showed that vonoprazan‐based triple therapy is superior to conventional PPI‐based triple therapy and that tegoprazan‐based therapy is noninferior to vonoprazan‐based regimens [16]. In that analysis, P‐CAB–based regimens were associated with higher eradication rates than PPI‐based regimens, with risk ratios of 1.13 in randomized controlled trials and 1.17 in observational studies. These findings indicate that P‐CAB–containing regimens are increasingly being adopted and may become first‐line therapy in many clinical settings.

In this context, the clinical role of molecular diagnostic testing should be reconsidered. Rather than being viewed merely as a diagnostic tool that remains reliable under acid‐suppressive therapy, Smart Gene may play an important role in optimizing P‐CAB–based eradication strategies by enabling the pre‐emptive identification of CAM resistance. As vonoprazan‐based regimens achieve high overall eradication rates, resistance‐guided antimicrobial selection may shift from a rescue strategy to a means of refining initial treatment choices. Such an approach has the potential to further enhance eradication success while avoiding unnecessary antibiotic exposure, even within highly effective P‐CAB–based regimens. This approach could also help avoid the burden of return visits for patients and help reduce healthcare costs. Importantly, the present study was not designed to evaluate eradication outcomes with vonoprazan‐based regimens; therefore, our data cannot determine whether resistance‐guided treatment using Smart Gene improves eradication rates under such regimens.

In this regard, the pooled eradication rates reported by Kanu and Soldera [16] provide an important benchmark against which the effectiveness of future Smart Gene–guided strategies, particularly those incorporating P‐CAB–based therapy, should be evaluated.

This study has some limitations. First, real‐time PCR was used as the reference standard. Although real‐time PCR is a widely accepted and clinically appropriate method for detecting *H. pylori* infection and CAM resistance, it is not a true gold standard and may yield false‐negative results. Indeed, one case in this study showed a negative real‐time PCR result but a positive Smart Gene result, suggesting a possible false‐negative PCR finding. Second, the sample size of patients with *H. pylori* infection was small owing to the low *H. pylori* infection rate. Given the exploratory nature of this study and the small number of *H. pylori*–positive cases (*n* = 23), no formal sample size or power calculation was performed, and the statistical power for subgroup analyses was limited. Consequently, the study was not adequately powered to support definitive subgroup comparisons, including those based on PPI/P‐CAB use, timing of intragastric fluid collection, or eradication outcomes stratified by resistance status. In addition, the effects of PPI/P‐CAB medication and the timing of intragastric fluid collection were not compared in the same patients. The type of PPI/P‐CAB and the duration of administration of these drugs were not investigated, and further detailed research is needed. However, this study demonstrated that the Smart Gene test results, a molecular diagnostic method, were less affected by PPI/P‐CAB medication and could be a useful method for patients requiring the management of gastrointestinal symptoms.

## 5. Conclusions

The Smart Gene *H. pylori* G molecular diagnostic kit is safe for sample collection and provides *H. pylori* infection and CAM‐resistance mutations within 1 h after safely collecting intragastric fluid during EGD. Moreover, the Smart Gene showed high diagnostic performance and was minimally affected by PPI/P‐CAB medication. Using this method could allow for the selection of an appropriate eradication treatment on the day of EGD, resulting in fewer unnecessary eradication treatment‐related side effects, a lower patient burden, and fewer resistant strains.

## Funding

This study was supported by Mizuho Medy Co. Ltd.

## Conflicts of Interest

The authors declare no conflicts of interest.

## Data Availability

The data that support the findings of this study are available from the corresponding author upon reasonable request. The data are not publicly available due to institutional and ethical restrictions.
